# Pragmatic trials: ignoring a mediator and adjusting for confounding

**DOI:** 10.1186/s13104-019-4188-1

**Published:** 2019-03-20

**Authors:** Paraskevi Pericleous

**Affiliations:** Data Science Campus, Office for National Statistics, Newport, UK

**Keywords:** Mediator, Confounder, Heterogeneity, Pragmatic, Trials

## Abstract

**Objectives:**

In pragmatic trials, the new treatment is compared with usual care (heterogeneous control arm) that makes the comparison of the new treatment with each treatment within the control arm more difficult. The usual assumption is that we can fully capture the relations between different quantities. In this paper we use simulation to assess the performance of statistical methods that adjust for confounding when the assumed relations are not true. The true relations contain a mediator and heterogeneity with or without confounding, but the assumption is that there is no mediator and that confounding and heterogeneity are fully captured. The statistical methods that are compared include multivariable logistic regression, propensity score, disease risk score, inverse probability weighting, doubly robust inverse probability weighting and standardisation.

**Results:**

The misconception that there is no mediator can cause to misleading comparative effectiveness of individual treatments when a method that estimates the conditional causal effect is used. Using a method that estimates the marginal causal effect is a better approach, but not for all scenarios.

**Electronic supplementary material:**

The online version of this article (10.1186/s13104-019-4188-1) contains supplementary material, which is available to authorized users.

## Introduction

Randomised controlled trials are held in an artificial environment with a carefully selected type of patients and placebo as the control group to the new treatment [1–3]. Pragmatic trials are held in real clinical practice, compare new treatment with usual care which consists of multiple treatments (heterogeneous control group) [4–6]. Comparing the new treatment with the usual care can be unbiased due to the randomization, but this is not true when comparing the new treatment with individual control treatments. Heterogeneity in the patient control group, which could lead to confounding, makes the comparison more complex. This paper considers a pragmatic trial, and thus there is heterogeneity in the control arm.

The usual assumption when dealing with pragmatic trials is that heterogeneity and confounding are fully captured or that the treatment causes the outcome [1]. The aim of this paper is to examine what happens when this assumption that is perceived as true, is false. The scenarios considered examine whether the treatment does not directly cause the outcome, but via a mediator in cases where heterogeneity and confounding are fully and partially captured. Different methods are compared for adjusting for measured confounding when there is the mistaken assumption that there is no mediator when heterogeneity with or without confounding are fully or partially captured. We will investigate this via simulations using multivariable logistic regression (Logistic), propensity score (PS), disease risk score (DRS), inverse probability weighting (IPW), doubly robust inverse probability weighting (DRIPW) and standardization (ST) [7, 8]. These methods are widely used and estimate the potential outcome if all the patients were on the same treatment [7]. The effect that the treatment has on the potential outcome is called causal effect, if it comes from a conditional model is called ‘conditional causal effect’ and if it comes from a marginal model is called ‘marginal causal effect’. Estimating the causal effects requires exchangeability which is ensured by randomization and collapsibility which is ensured by adjusting for a fully captured confounding [7, 9].

The motivation for using a multivariable logistic regression is because it is widely used when the interest is to predict a binary outcome, e.g. whether the patient is dead or alive, hospitalized or not, cancer metastasis or not within a specific time-period. A recent example of that can be found in Agarwal et al. [10] where they used hospital admission as a binary outcome with a logistic regression model.

## Main text

### Methods

We use the same notation as in Pericleous et al. [1]. Z is the treatment allocation (Z = 0, 1, 2 denote the new treatment, baseline control treatment and second control treatment respectively). $n\, = \,\sum\nolimits_{k = 0}^{2} {n_{k} }$, is the total number of patients participating and $n_{k}$ the number of patients within each treatment group. Y, C ~ Bernoulli (0.5) and U ~ $N\,\left( {0,0.64} \right)$ are the binary outcome, observed and unobserved heterogeneity. We use no intercepts for the logit models to simplify the models. Assuming no refusals, patients are assigned to usual care using:

1 $${\text{logit}} \left( {P\left[ {Z\, = \,2|C,U} \right]} \right)\, = \,\alpha_{1} C\, + \,\alpha_{2} U\, + \,\alpha_{3} CU,\quad (Z\, = \,1\,{\text{otherwise}})$$

where $I\left[ . \right]$ is the indicator function. We assumed a linear relationship of the mediator depending on the treatment:

2 $${\text{M | V, }}Z\, = \, \beta_{0} V\, + \, \beta_{1} I\left[ {Z\, = \,0} \right]\, + \,\beta_{2} I\left[ {Z\, = \,2} \right],\quad {\text{ where V}}\sim N\left( {0,1} \right)$$

The binary outcome (alive or dead) Y is given by:

3 $${\text{logit}} \left( {Y|M,U} \right)\, = \,\beta_{3} C\, + \, \beta_{4} U\, + \,\beta_{5} M .$$

We are interested in the scenarios shown in Table 1. More details for the scenarios are shown in Additional file 1: Figures S1 and S2. Additional file 1: Figure S1 shows the true relations, while Additional file 1: Figure S2 shows the assumption made. We want to examine the asymptotic behaviour of the models and thus, we use 10,000 patients and 1000 replications for each scenario. Having 10,000 patients in a pragmatic trial is a rare scenario. However, choosing a large number for patients and replication we ensure that we examine the asymptotic properties of the models and that the results are not due to chance. We need to clarify that if the asymptotic behaviour of the model is problematic and cannot provide unbiased results, then having a small number of patients will not change that. We are mainly interested in estimating $\beta_{1}$ and $\beta_{2}$.

**Table 1:** Simulation scenarios

Scenario	Parameter settings
(a) No unmeasured heterogeneity or confounding	α_2_ = α_3_ = β_5_ = 0, α_1_ = β_0_ = β_1_ = β_2_ = β_4_ = β_6_ = 1
(b) Unmeasured heterogeneity with confounding	α_1_ = α_2_ = α_3_ = β_0_ = β_1_ = β_2_ = β_4_ = β_5_ = β_6_ = 1
(c) Unmeasured Heterogeneity without confounding	α_2_ = α_3_ = β_5_ = 0, α_1_ = β_0_ = β_1_ = β_2_ = β_4_ = β_6_ = 1

We applied the most widely used methods for adjusting for confounding, as in Pericleous et al. [1]. These include the ones that calculate the conditional causal effect: (1) multivariable logistic regressions adjusted for confounding; (2) propensity score (PS), (3) disease risk score adjustment (DRS), (4) doubly robust inverse probability weighting (DRIPW), and the ones that calculate the marginal causal effect, (5) inverse probability weighting (IPW) and (6) standardisation [1, 7, 8].

PS is the probability to receive a specific treatment given the observed covariates using a multivariable logistic regression. Then, used as a covariate or as weights to predict the binary outcome [8]. In our case, we used it as a covariate. DRS is the probability of the binary outcome using a logistic regression with the treatment as a covariate. Then, used as a covariate to predict the binary outcome [8]. IPW uses weights which calculates by dividing the probability of the observed treatment exposure with the probability of the binary outcome using a Logistic regression given the confounders [7]. DRIPW uses the IPW weights to model the outcome, but the confounders are also used on the same model [7]. Standardisation: expands the dataset, modelling the outcome, getting the prediction and standardizing by averaging [7] (standardizes the mean outcome to the confounder distribution). It is mathematically equivalent to IPW [7]. For more details on the methods see Hernan and Robins [7], Pericleous et al. [1] and Schmidt et al. [8].

### Results

The first four methods estimate the conditional causal effect (Logistic, DRIPW, PS, DRS) and the final two methods estimate the marginal causal effect (IPW and ST). All methods used are based in the misconception that there is no mediator. In Scenario 1, the methods that estimate the conditional causal effect do not perform well and provide biased results. The methods that estimate the marginal causal effect provide unbiased estimates of both $\beta_{1}$ and $\beta_{2}$ (Additional file 1: Figure S3). In Scenario 2, it seems that partially captured confounding leads to biased results from all methods (Additional file 1: Figure S4). Mathematically, not considering a mediator that exists creates a partially captured heterogeneity, and thus Scenario 3 could be considered as having two different kind of heterogeneity. This is the possible reason why all methods (except ST that is doing slightly better in Scenario 1) perform relatively the same in Scenario 1 and Scenario 3 (Additional file 1: Figure S5).

### Discussion

Ignoring a mediator and adjusting for confounding lead to biased estimates of the conditional causal effect, even though the heterogeneity and confounding are fully captured. IPW and Standardisation, however, provide unbiased estimates of the marginal causal effect under these circumstances. In cases where there is unobserved heterogeneity standardization is not as good as IPW in estimating the marginal causal effect, but it provides a rather good estimate. In cases, however, where confounding and heterogeneity are not fully captured all the methods provide biased results for both the conditional and the marginal causal effect. In conclusion, ignoring a mediator can lead to misleading conclusions about the comparative effectiveness of individual treatments when using methods that estimate the conditional causal effect. It is advised to use methods that calculate the marginal causal effect such as IPW that provide unbiased estimators when ignoring a mediator. However, in the case where unobserved heterogeneity and confounding exist then all the methods provide biased estimates.

## Limitations

The limitations in this study are: we assume no treatment refusals, that there is only one mediator and that the relationship between the mediator and the treatments is linear.

## Additional file

**Additional file 1.** Additional figures.
