# Supplementary material for: Pragmatic trials: ignoring a mediator and adjusting for confounding
Source: BMC Res Notes. 2019 Mar 20;12:156. doi: 10.1186/s13104-019-4188-1 (PMC6425675; doi:10.1186/s13104-019-4188-1)
Supplement: Supplementary file 1 — Additional file 1. Additional figures. [file 13104_2019_4188_MOESM1_ESM.docx]

File Name: Supplementary Material

Title of data: Ignoring a Mediator and Adjusting for Confounding Data

Description of data: The data was created with treatment to be affecting a Mediator which was then affecting the binary outcome. This data was then used to examine if there is a bias when the usual assumption is performed in the analysis, that the treatment directly affects the outcome.

C Z C Z C Z

Y Y U Y U

M M M

(a) (b) (c)

**Figure S1.** Causal diagrams for the true relations in the three scenarios. C, Z, Y, U and M denote the observed confounder, treatment, binary outcome, unobserved heterogeneity and mediator respectively. Each panel represents each scenario mentioned in Table 1.

C Z C Z C Z

Y Y U Y U

1. (b) (c)

**Figure S2.** Causal diagrams for the assumed relations in the three scenarios. C, Z, Y and U, denote the observed confounder, treatment, binary outcome and unmeasured heterogeneity. Each panel represents each scenario mentioned in Table 1.


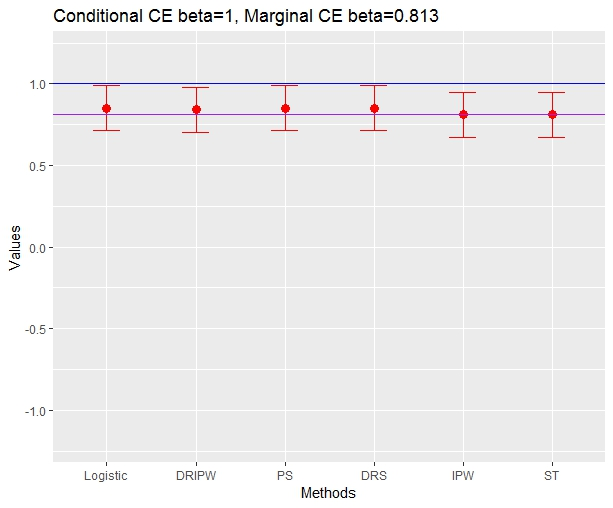

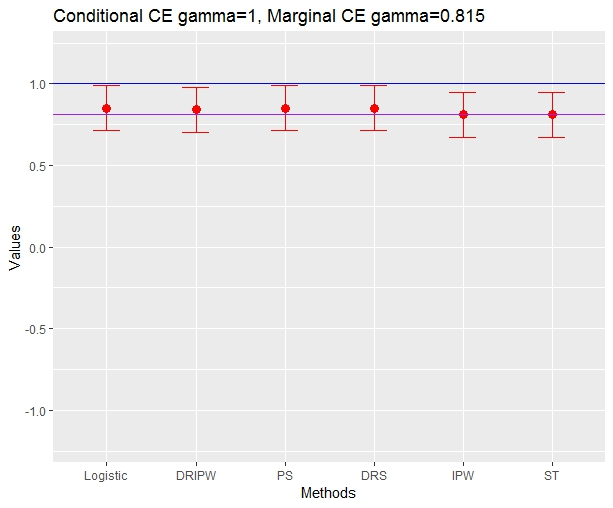


**Figure S3.** Estimates of conditional and marginal causal effects of the estimates of β_1_ (left) and β_2_ (right) for Scenario 1. Red: 95% confidence interval (CI) of estimates; blue: true conditional causal effect; purple: true marginal causal effect.


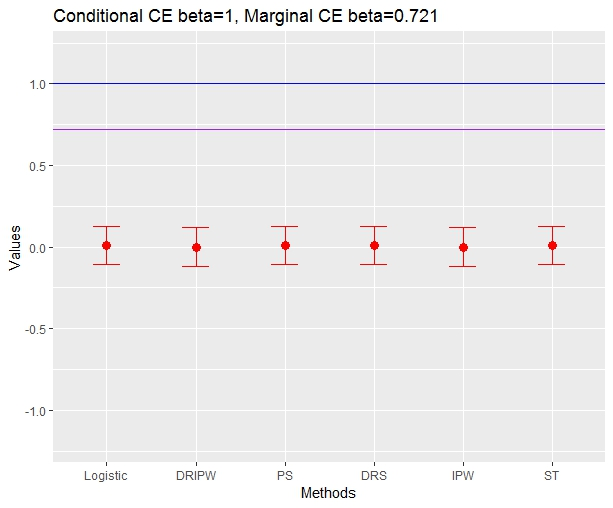

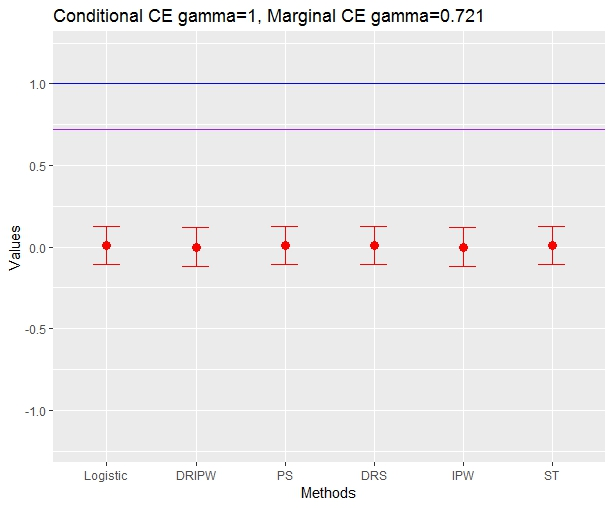


**Figure S4.** Estimates of conditional and marginal causal effects of the estimates of β_1_ (left) and β_2_ (right) for Scenario 2. Red: 95% confidence interval (CI) of estimates; blue: true conditional causal effect; purple: true marginal causal effect.


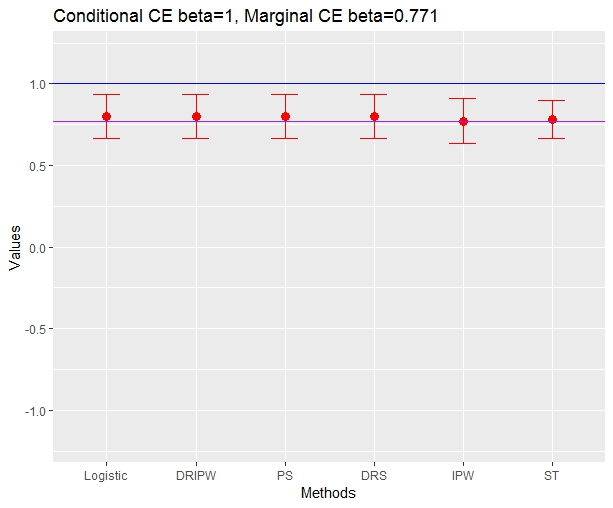

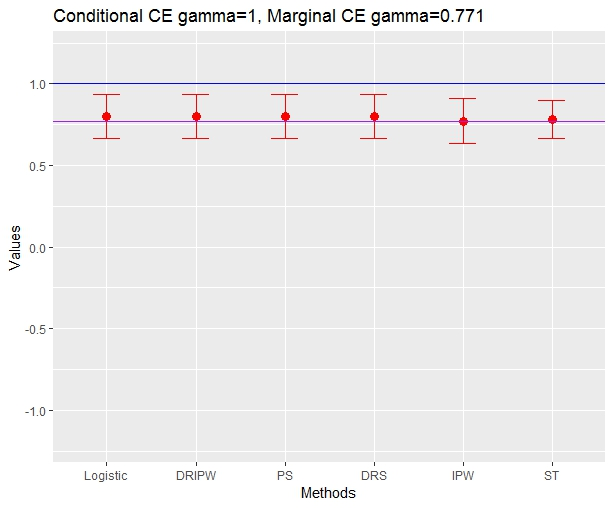


**Figure S5.** Estimates of conditional and marginal causal effects of the estimates of β_1_ (left) and β_2_ (right) for Scenario 3. Red: 95% confidence interval (CI) of estimates; blue: true conditional causal effect; purple: true marginal causal effect.
